# Pharmacological Activity of Quercetin: An Updated Review

**DOI:** 10.1155/2022/3997190

**Published:** 2022-12-01

**Authors:** Guanzhen Wang, Yuanhui Wang, Liangliang Yao, Wei Gu, Shengchao Zhao, Ziyi Shen, Zihan Lin, Wei Liu, Tingdong Yan

**Affiliations:** ^1^University and College Key Lab of Natural Product Chemistry and Application in Xinjiang, School of Chemistry and Environmental Science, Yili Normal University, Yining 835000, China; ^2^School of Life Sciences, Shanghai University, 99 Shangda Road, Shanghai 200444, China; ^3^Affiliated Hospital of Jiangxi University of Chinese Medicine, Nanchang 330006, China

## Abstract

Quercetin, a natural flavonoid compound with a widespread occurrence throughout the plant kingdom, exhibits a variety of pharmacological activities. Because of the wide spectrum of health-promoting effects, quercetin has attracted much attention of dietitians and medicinal chemists. An updated review of the literature on quercetin was performed using PubMed, Embase, and Science Direct databases. This article presents an overview of recent developments in pharmacological activities of quercetin including anti-SARS-CoV-2, antioxidant, anticancer, antiaging, antiviral, and anti-inflammatory activities as well as the mechanism of actions involved. The biological activities of quercetin were evaluated both *in vitro* and *in vivo*, involving a number of cell lines and animal models, but metabolic mechanisms of quercetin in the human body are not clear. Therefore, further large sample clinical studies are needed to determine the appropriate dosage and form of quercetin for the treatment of the disease.

## 1. Introduction

**Figure 1:**
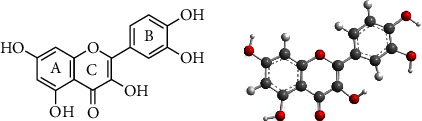
Chemical structure and 3D conformer of quercetin.

Polyphenols are naturally occurring chemical compounds in plants. Generally, polyphenols, comprising more than 8000 compounds, are divided into 2 main groups: flavonoids and non-flavonoids (phenolic acids, lignans, stilbenes, nonphenolic metabolites, and other polyphenols) [1]. The flavonoid is composed of three benzene rings and five hydroxyl groups. Quercetin [2-(3,4-dihydroxyphenyl)-3,5,7-trihydroxy- 4H-chromen-4-one] has 2 benzene rings (*A* and *B* rings) that are connected by a 3-carbon chain to form a closed pyran ring (C ring, Figure 1). There are a total of 5 hydroxyl groups, and glycosylation can occur on any hydroxyl group, producing various quercetin glycoside forms by binding to glucose, xylose, or rutin sugar [2]. Furthermore, the dihydroxy group between the A ring, the *o*-dihydroxy group of the B ring, ∆^2(3)^ and 4-carbonyl of the C ring are the active groups present in quercetin. The biological activity of quercetin is largely attributed to these active phenolic hydroxyl groups and double bonds [3]. *B*-ring in the class of natural flavonoids is the main active site for antioxidant and reactive oxygen species (ROS) scavenging [4]. Flavonoids also play an important role in platelet aggregation, the peroxidation of lipids, and enhancing the biogenesis of mitochondria [5].

**Figure 2:**
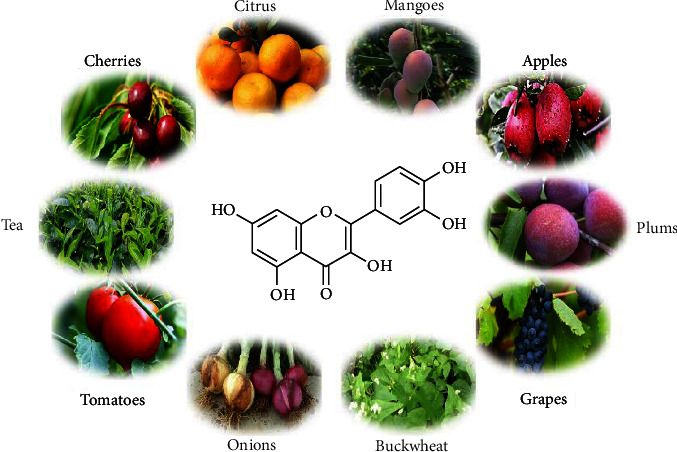
Sources of quercetin.

Quercetin is a plant secondary metabolite that occurs widely in different parts of the plant and is also a basic component of the human diet [5]. The richest source of quercetin is onion, one of the most popular vegetables, both edible and medicinal [3]. Other sources include grapes, cherries, apples, mangoes, citrus fruits, buckwheat, plums, tomatoes, and tea (Figure 2) [6, 7].

The steps in which quercetin is oxidized are as follows: the phthalone fraction is first oxidized, the benzofuranone derivative is formed by an intramolecular rearrangement mechanism, the benzofuranone is subsequently oxidized, and then, the resorcinol structure is oxidized [8]. Quercetin and its derivatives possess multiple pharmacological activities including anti-SARS-CoV-2, antioxidant, anticancer, antiaging, antiviral, and anti-inflammatory properties [6, 9]. Quercetin is known to use in the treatment of cancer, allergic reactions, inflammation, arthritis, and cardiovascular disorders [3, 9]. Reportedly, quercetin might be a potential therapeutic candidate against epilepsy that deserves further investigation [10]. Quercetin has a promising therapeutic effect on sepsis complications [11]. In addition, quercetin possesses neuropharmacological protective effects against neurodegenerative brain disorders such as Alzheimer's disease, Parkinson's disease, Huntington's disease, amyloid *β* peptide, multiple sclerosis, and amyotrophic lateral sclerosis [12]. Quercetin develops the potential of alternative or complementary medicine in atherosclerosis [6]. Pyruvate dehydrogenase kinase 3 is a mitochondrial protein that has recently been considered a potential pharmacological target for the treatment of varying types of cancer. Dahiya et al. [13] found that quercetin has a significant inhibitory effect on pyruvate dehydrogenase kinase 3(PDK3) with the IC_50_ values in *μ*M range. Thus, quercetin may be further evaluated as a promising therapeutic molecule for PDK3 with required modifications and *in vivo* validation. Furthermore, quercetin derivatives present in systemic circulation after consumption of quercetin may act as a potent antioxidant and anti-inflammatory agents and can contribute to the overall biological activity of a quercetin-rich diet [14]. In the past decades, a series of natural and synthetic compounds have been used in the clinical treatment of various diseases due to their good pharmacological activities. For example, saponins as plant-derived natural products are proved to exert their physiological activities through binding to nuclear receptors, making them promising candidates for selective receptor modulators [15, 16]. Quercetin shows greater potential for clinical use in the future. Therefore, the pharmacological activities of quercetin and its mechanisms of action are reviewed in this paper.

## 2. Anti-SARS-CoV-2 Activity

Severe acute respiratory syndrome coronavirus-2 (SARS-CoV-2) (COVID-19) represents an emergent global threat that is straining worldwide healthcare capacity. As of August 18th, the disease caused by SARS-CoV-2 has resulted in more than 6,400,000 deaths worldwide, with 1,060,000 deaths in the US alone. Quercetin, as the main effective therapeutic ingredient in traditional Chinese medicine, may effectively treat and prevent COVID-19 [17]. Epidemic infectious diseases have always been a significant problem plaguing human progress. In particular, the SARS-CoV-2 pandemic is a massive challenge for many poorer countries lacking specialized equipment and laboratories. To address this crisis, developing biosensors with rapid, easy, and non-device-dependent detection is paramount. In our previous review, an increasing number of studies have shown that CRISPR/Cas technology can be integrated with biosensors and bioassays for nucleic acid detection. In addition, it is particularly important to develop drugs for clinical treatment [18]. Quercetin can interfere with SARS-CoV-2 replication theoretically as well as reduce the inflammation and toxic effects of coronavirus disease 2019 (COVID-19) vaccines [19, 20]. Moreover, quercetin shortens the timing of molecular test conversion from positive to negative while reducing at the same time symptoms severity and negative predictors of COVID-19 [21]. In a recent clinical study, the researchers divided 42 COVID-19 symptom outpatients into two groups with one group receiving standard care (SC) treatment and the other group receiving quercetin as a supplemental treatment based on this. By blood indicators, the addition of quercetin supplementation reduced lactate dehydrogenase (LDH) (−35.5%), ferritin (FER) (−40%), C-reactive protein (CRP) (−54.8%), and D-dimer (−11.9%) in the patient's blood. Quercetin supplementation not only shortens the time it takes for molecular experiments to turn positive to negative but also reduces the severity of symptoms of COVID-19 [21]. The combination of quercetin and vitamins can be used for the treatment of COVID-19 patients [22]. Quercetin may be an effective intervention to decrease the frequency and duration of respiratory tract infections; however, more research is needed [23].

## 3. Antioxidant Activity

Oxidative stress refers to the pathophysiological responses caused by excessive production of highly reactive molecules such as ROS in the body and the dysregulation of the oxidant and antioxidant balance in the body when subjected to various harmful stimuli. Oxidative stress can cause mitochondrial DNA damage, intracellular protein denaturation, lipid peroxidation and inflammation, and apoptosis or necrosis of cardiomyocytes [24]. Due to the phenolic hydroxyl group and the presence of a double bond, quercetin owes potential antioxidant activities. Quercetin is a potent scavenger for ROS and hence protects the body against oxidative stress. In addition, quercetin maintains the oxidative balance and hence is a strong antioxidant, and it regulates the glutathione (GSH) level in the body [25]. Studies of animals and cells have shown that the synthesis of GSH is induced by quercetin. The increased expression of superoxide dismutase (SOD), catalase (CAT), and GSH has been reported with the pretreatment of quercetin. Numerous studies have shown that quercetin interacts directly with DNA and that quercetin covalently binds to DNA [25]. It is uncertain whether quercetin repairs DNA or protects it from oxidative damage.

Quercetin could be used as a safe dietary polyphenol to inhibit lipid oxidation [26]. Quercetin markedly reduced the production of ROS in the microglia, and it enhanced the M2 macrophage polarization and endogenous antioxidant expression in both macrophages and microglia [27]. It has been reported that the esterification reaction does not affect the antioxidant activity of quercetin. Quercetin inhibited aldehyde formation and unsaturated fatty acid oxidation in fish oil significantly [28]. Therefore, quercetin derivatives might be useful as possible antioxidants in food and biological systems.

Arslan et al. [29] found that quercetin supplementation to layer chickens significantly reduced malondialdehyde (MDA) levels in the kidneys, liver, and heart and increased GSH, CAT, and glutathione peroxidase (GSH-Px) activities in the liver, kidney, and heart tissues. Therefore, dietary supplementation with quercetin can alleviate oxidative stress, biochemical changes, and apoptosis in quail. In addition, ultraviolet radiation *b* (UVB) induces intracellular ROS production in HaCaT cells, and quercetin has been found to be effective in inhibiting UVB-induced intracellular ROS production, thereby protecting mitochondria [30]. Saw et al. [31] made a model of oxidative stress of HepG2-C8 cells, and after pretreatment with quercetin, it was found by DPPH analysis that quercetin had strong oxygen radical scavenging activity, and the results of DCFH-DA determination and combined index also confirmed that quercetin can reduce the level of reactive oxygen species in cells under oxidative stress. In addition, quercetin can exert antioxidant effects by chelating Cu^2+^ and Fe^2+^ in its structure with catechol [32].

Increased apoptosis induced by oxidative stress is considered as an important pathological change in seminal vesicles in patients with diabetes. Dong et al. [33] studied the effect of quercetin on apoptosis of seminal vesicle cells and its mechanism, established a type 1 diabetic rat model induced by streptozotocin, and administered quercetin in three doses of low, medium, and high doses for 4 months. Fasting blood glucose, refined fructose, total vesicle antioxidant capacity (T-AOC), and MDA levels were measured, as well as the expression of nuclear transcription factor 2(Nrf2), apoptosis-related biomarkers B-cell lymphoma 2 protein (Bcl2), Bcl2-associated*x* protein (Bax), and caspase-3. The results showed that T-AOC and Nrf2 decreased, malondialdehyde levels increased, caspase-3 cleavage increased, Bax/Bcl2 ratio decreased, and accompanied by severe hyperglycemia in seminal vesicles of diabetic rats. After 4 months of treatment, except for fasting blood glucose, the remaining indicators were reversed to varying degrees. Therefore, quercetin may alleviate apoptosis of seminal vesicle cells caused by oxidative stress by inhibiting Nrf2, suggesting that it can be used to prevent damage to seminal vesicles in rats with type 1 diabetes [33].

## 4. Anticancer Activity

### 4.1. Antitumour

Cancer is a highly malignant disease. Based on its current status, it is urgent to explore a kind of drug with lower toxicity, lower side effects, and effective drug for cancer treatment or adjuvant therapy. The tumor occurrence and development involve multiple pathways, multiple links, and multiple targets [34]. The complexity of interactions between the various links can lead to clinical responses, such as limited therapeutic effects and larger side effects [35]. Recently, intense attention has been paid to the application of natural compounds as a novel therapeutic strategy for cancer treatment. As a natural product, quercetin is a flavonoid compound that is nontoxic when treated with a reasonable dose of quercetin and has various inhibitory effects on various ways of tumor formation [34]. The anticancer mechanism of quercetin is mainly through inhibiting cancer cell proliferation, inducing apoptosis, inducing autophagy of cancer cells, regulating signaling pathways, inhibiting its invasion and metastasis, enhancing chemotherapy sensitivity, and reversing drug resistance [36–38]. Available experimental studies indicate that quercetin could modulate multiple cancer-relevant miRNAs including let-7, miR-21, miR-146a, and miR-155, thereby inhibiting cancer initiation and development [9]. Quercetin treatment combined with the functions of growth factor midkine knockdown strategy could potentially target CD44+/CD133+ cells and promote elimination, thereby preventing cancer relapse [39].

Quercetin can influence the development of tumor by regulating epigenetics, which can directly regulate the expression of miRNA and the level of DNA methylation to exert an anticancer effect and enhance the sensitivity of tumor cells to chemotherapy [36]. Quercetin has a significant antitumor effect in osteosarcoma [40]. There is evidence that the possibility of using quercetin as a therapeutic option for glioblastoma multiforme [41]. Quercetin and kaempferol inhibit rhabdomyosarcoma cell and tumor growth [42]. The study found that quercetin possesses antitumor effects on malignant cells and induces its anticancer effect against cancer cells via modulating various signaling pathways involved in cancer development and progression. Quercetin inhibits the proliferation of liver cancer cells via induction of apoptosis and cell cycle arrest [43]. Quercetin exhibits direct proapoptotic effects on tumor cells and thus can inhibit the progress of numerous human cancers [34].

Among various natural compounds, quercetin has shown great anticancer and anti-inflammatory properties. Vafadar et al. [44] found that experiments have revealed that quercetin possesses a cytotoxic impact on ovarian cancer cells *in vitro* and *in vivo*. Studies have shown that quercetin induces apoptosis, inhibits metabolic activity, and cell death in hepatocellular carcinoma cells (HepG2, HuH7) [45]. Quercetin suppresses hepatoblastoma cell proliferation and invasion and promotes apoptosis [46, 47]. Quercetin could disturb LM3 cells proliferation and cell cycle distribution and inhibit LM3 cells migration and invasion, thus inducing apoptosis and promoting hepatocellular carcinoma autophagy. [48] In cell culture and rodent studies, the mechanism of action and targets of quercetin are mainly involved in Wnt/*β*-catenin, MAPK/ERK, MAPK/JNK, PI3K/AKT/mTOR, MAPK/p38, p-53, and NF-*κ*B. [38] Besides, quercetin can be used as chemoprophylaxis and optionally in combination with chemotherapeutic drugs to improve clinical outcomes in patients with prostate cancer. [34] Quercetin in PA-1 human ovarian cancer cell line significantly reduces cell viability by a dose-dependent manner, enhances apoptosis of aggressive ovarian cancer cell lines, reduces Bcl-2 and Bcl-xL, increases Bad, Bid, Bax, caspase-3, caspase-9, and cytochrome C, and enhances the mitochondrial-induced pathway of apoptosis, thereby inhibiting the growth of invasive ovarian cancer cells [49]. Quercetin acts at a concentration of 5 *μ*M in H1975 and A549 human lung cancer cell lines, which significantly inhibits nickel-mediated invasion of H1975 and A549 lung cancer cells, inhibits inflammatory mediator secretion, inhibits mRNA and protein expression of TLR4 and Myd88, reduces phosphorylation of IKK*β* and I*κ*B, reduces expression of NF-*κ*B and matrix metalloproteinase (MMP)-9, and induces inactivation of the TLR4/NF-*κ*B signaling pathway [50]. In HSC-6, SCC-9 human oral cancer cell lines, quercetin inhibits cell viability, migration, and invasion, reduces MMP-2 and MMP-9 abundance, downgrades miR-16, and upgrades HOXA10 [51]. Quercetin reduces invasion, adhesion, proliferation, and migration of human metastatic osteosarcoma cells by inhibiting the expression of parathyroid hormone receptors 1 and MMP-2 and MMP-9 [52].

### 4.2. Cancer Prevention

Cancer chemoprevention is a prevention strategy that involves the long-term use of one or more natural or synthetic drugs to block or inhibit the course of cancer before it becomes an aggressive disease. Quercetin is an ideal treatment tool for chemoprevention of cancer, specifically activating apoptosis of cancer cells without inhibiting the cell growth of normal cells [53]. Quercetin can be used as a supplement for cancer prevention and as a low-toxicity therapeutic molecule for cancer treatment [54]. According to existing research, quercetin is considered to be a complementary or alternative medicine for the prevention and treatment of different cancers. At appropriate molar ratios of quercetin and curcumin, proliferation and apoptosis of the human breast cancer cell line MCF7 were significantly reduced [55]. In addition, quercetin inhibits the mobility of cancer cells by inhibiting glucose uptake and lactic acid production and reducing levels of PKM2, GLUT1, and LDHA, which may have a significant role in controlling breast cancer [56].

### 4.3. Anticancer Agent

Quercetin combined with a variety of small molecule drugs can reduce the dosage of anticancer agents and improve the overall efficacy and safety by regulating signal molecules and blocking cell cycle [11]. Quercetin and cisplatin have a synergistic inhibitory effect on cervical cancer cells [57]. Quercetin might enhance the antitumor effect of cisplatin via inhibiting proliferation, migration, and invasion and elevating apoptosis through weakening MMP-2, ezrin, METTL3, and P-Gp expression of cancer cells. Sunoqrot et al. [58] loaded the plant polyphenol quercetin with hydrophilic anticancer curcumin and functionalized it with poly-(ethylene glycol) for drug delivery applications of cancer cell lines with higher antiproliferative properties. In this study, quercetin-based nanomaterials were observed to have a spherical form in most cases and good solubility in aqueous solutions, which clearly enhances their anticancer effectiveness. Furthermore, the effects of quercetin on glucose metabolism and cellular energy production contribute to its effect on cell viability reduction, metastasis inhibition, and apoptosis induction in cancer cells [35].

Quercetin nanoparticles have shown high encapsulation efficiency, stability, sustained release, prolonged cycle time, improved accumulation at tumor sites, and therapeutic efficiency. The combination of quercetin with other diagnostic or therapeutic agents in nanocarriers has achieved enhancement in the detection or treatment of tumors [59]. Since quercetin is insoluble in water and more difficult to dissolve in alcohol, Ezzati et al. [60] studied quercetin using organic solvent dissolution methods. They prepared nanoparticle preparations that were improved as a whole anticancer agent when quercetin was wrapped in a poly lactic-co-glycolic acid nanoparticle system. A large number of *in vivo* and *in vitro* experiments have shown that quercetin has a strong role in promoting apoptosis, inhibiting metastasis, and its ability to regulate cell cycle and tumor angiogenesis (Table 1).

## 5. Antiaging Activity

Cellular senescence is a state of irreversible cell cycle arrest, which can be induced by a variety of stressors, including telomere dysfunction and genotoxic and oxidative stress [67]. Aging cells lead to age-related tissue degeneration, and the accumulation of senescent cells promotes fat accumulation and steatosis in the liver. Recent studies have shown that in INK-ATTAC transgenic mice, treatment with a combination of quercetin and dasatinib not only eliminates senescent cells but also reduces overall liver steatosis [68].

Slowing the progression of aging may be an attractive way to preserve islet function after transplantation. In terms of quercetin's effect on improving islet transplant results, Pathak et al. [69] established a model of *in vitro* induced premature aging of rat islets, transported quercetin to the islets in situ by using polymer microspheres, and then constructed a hybrid cluster of islets and microspheres by suspension method, and in long-term*in vitro* culture, the presence of quercetin in the cell microenvironment slowed down the aging process of islets. In addition, transplanting hybrid clusters into diabetic mice produced better glycemic control compared to control islets. Therefore, topical injections of antioxidants, such as quercetin, may be an attractive way to improve cell therapy outcomes. Quercetin can reduce liver failure caused by bile duct ligation (BDL) or carbon tetrachloride (CCl_4_), can inhibit the formation of extracellular matrix, and can regulate MMP-9 and metalloproteinase tissue inhibitors (TIMP)-1. Quercetin prevents liver failure by inhibiting the TGF-*β*1/Smads signal pathway, activating the PI3K/AKT signal pathway, and inhibiting autophagy in BDL or CCl_4_tetrachloride-induced liver failure [70].

While cellular senescence may be a protective mechanism that regulates proliferative capacity, fibroblast senescence is now considered a key pathogenic mechanism for idiopathic pulmonary fibrosis. Quercetin alone can eliminate resistance of idiopathic pulmonary fibrosis fibroblasts to Fas ligands (FasL) or trial-induced apoptosis. Quercetin reverses the resistance to death ligand–induced apoptosis by promoting FasL receptor and caveolin-1 expression and inhibiting AKT activation, thus mitigating the progression of established pulmonary fibrosis in aged mice [71]. Therefore, quercetin may be a viable treatment for idiopathic pulmonary fibrosis and other age-related diseases. In addition, chronic clearance of senescent cells using quercetin can improve renal functional indices and alleviated fibrosis [72].

In a recent report on the effect of quercetin on the tenderness of chicken pectoral muscles and its related mechanisms, quercetin can significantly reduce shear force and increase myofiber rupture index. After quercetin treatment, Bip, caspase-3 activity, and p-IRE1/IRE1 and Bax/Bcl-2 ratios were improved, and caspase-12 was activated. In addition, quercetin can also induce cell transition from LC3I to LC3II and increase the expression of ATG7 and Beclin-1. The PI3K/AKT/mTOR signal pathway is involved in quercetin-induced autophagy and apoptosis. These results suggest that quercetin can promote meat tenderness and activate apoptosis and autophagy pathways during the aging process of chickens after death [73].

Liver fibrosis is one of the leading causes of death worldwide. Quercetin has a significant antifibrotic effect in liver fibrosis. Hepatic astrocytic cells are activated during chronic liver injury, expressing more transforming growth factors *β*, collagen 1*α*, and actin-*α*-smooth muscle, which leads to liver fibrosis, quercetin by downregulating the conversion of growth factor-*β*/Smad3 signal pathways, effectively reducing the expression of fibrosis genes in fructose-activated human liver stellate cells, thereby exerting liver protection [74]. In addition, quercetin inhibits the differentiation of mesenchymal progenitor cells into fat cells. Under the condition of adipocyte differentiation, quercetin-treated PDGFR*α*^+^/CD201^+^ cells inhibit fat deposition and expression of the lipid-forming genes CEBPA and ADIPOQ by inhibiting the phosphorylation of CREB. Quercetin significantly reduces the expression of probromine genes (TIMP1, ACTA2, COL1A1, and COL3A1) by inhibiting the phosphorylation of Smad2. Within the range of concentrations achievable with dietary and dietary supplement intake, quercetin inhibits the differentiation of muscle-derived PDGFR*α*^+^/CD201^+^ cells into fat cells and fibroblasts [75]. This suggests that quercetin has a preventive or therapeutic effect on the loss of muscle mass. Quercetin has been reported to play an antiaging role by selectively removing aging endothelial cells [76].

## 6. Antiviral Activity

Quercetin and its derivatives have antiviral effects on a variety of viruses, including human immunodeficiency virus (HIV), polio virus, respiratory virus, Sindbis virus, Mayar virus, and H5N1 virus [77]. Several studies highlight the potential use of quercetin as an antiviral drug due to its ability to inhibit the initial stages of virus infection, to interact with proteases important for viral replication, and to reduce inflammation caused by infection. Liu et al. [78] found that quercetin has a significant damaging effect on Singapore grouper iridovirus particles. Quercetin not only interfered with the binding of SGIV to host cell targets (76.14%) but also interfered with the virus's invasion of host cells (56.03%), affecting its replication within host cells (52.73%). Therefore, quercetin has a direct and host-mediatedanti-Singapore grouper iridescence virus and has the application prospect of developing effective drugs to control Singapore grouper iridescence virus infection in aquaculture. Quercetin and isoquercitrin are bioactive compounds in the ethyl acetate fraction of *Elaeocarpus sylvestris* that effectively inhibit human herpesvirus replication [79]. There is evidence that quercetin and vitamin C coadministration exerts a synergistic antiviral action due to overlapping antiviral drugs of ascorbic acid and immunomodulatory properties and the capacity of ascorbate to recycle quercetin, increasing its efficacy [22].

Quercetin has a preventive effect on plant virus infections. Wang et al. [80] optimized the prescription by adding a few of surfactants and stabilizers using biomaterials as raw materials to obtain 117 nm quercetin nanoliposomes with good stability. They found that Nbhsp70er-1 and Nbhsp70c-A were target genes for quercetin and that nanoliposomes had a 33.6% and 42% increase in inhibition at gene and protein levels, respectively. The results of field efficacy tests indicated that the efficacy of the agent was 38% higher than that of conventional preparations and higher than that of other antiviral agents. Therefore, the combination of biological antiviral agents and nanotechnology to control plant virus diseases has significantly improved the control efficiency and reduced the use of traditional chemical pesticides. Furthermore, quercetin antioxidant treatment may be helpful for preventing mycotoxin toxicity in food and feed industry [81].

## 7. Anti-Inflammatory Activity

Quercetin not only inhibited neutrophil infiltration but also promoted the apoptosis of activated neutrophils and reduced the plasma levels of inflammatory cytokines [82]. Therefore, it may be an alternative agent for the treatment of rheumatoid arthritis by inhibiting neutrophil activities. In terms of the protective effects of macrophage apoptosis, quercetin inhibits the expression of NLRP3 and lysate cysteine protease 1 in a concentration-dependent manner, as well as the expression of IL-1*β* and N-GSDMD, thereby preventing THP-1 macrophage apoptosis. Quercetin can inhibit the activation of NLRP3 inflammasomes. In addition, quercetin inhibits the elevation of TLR2/MyD88 and p-AMPK induced by lipopolysaccharide/adenosine triphosphate, and quercetin exerts an anti-inflammatory effect by inhibiting TLR2/MyD88/NF-*κ*B and ROS/AMPK pathways [83]. The combined use of quercetin and dasatinib can relieve intestinal aging and inflammation in older mice [84].

Quercetin can improve the degeneration of osteoarthritis by weakening the oxidative stress responses and inhibiting the degradation of cartilage extracellular matrix [85]. Quercetin treatment could inhibit inflammation-induced mitochondrial fission and promote mitochondrial fusion [24]. Activation or inhibition of the NLRP3 inflammasome is affected by regulators such as TXNIP, SIRT1, and Nrf2. Quercetin suppresses the NLRP3 inflammasome by affecting these regulators [86]. Quercetin has an inhibitory effect on inflammatory responses. On the other hand, it not only inhibits the production of NLRP3 inflammasome components and pro-IL-1*β* but also suppresses inflammation through interference in various signal pathways, especially NF-*κ*B. The NLRP3 inflammasome is affected by regulators such as TXNIP, SIRT1, and Nrf2, which play a key role in the activation or inhibition of the NLRP3 inflammasome. The quercetin also affects these factors, thereby suppressing NLRP3 inflammasome and eventually inflammation [86]. Quercetin decreased ROS-induced oxidative stress and inflammation by suppressing NOX2 production [87]. Treatment with quercetin effectively reduces the *M*1 inflammatory responses that stimulate NO production, proinflammatory cytokine expression, and lipocalin-2 production in both macrophages and microglial cells. The chemokines, C-C motif chemokine ligand (CCL)-2 and CCL-10, were also inhibited by quercetin treatment [24]. Numerous studies have shown that quercetin has anti-inflammatory activity both inside and outside the body (Table 2).

A recent study demonstrated the effect of quercetin in regulating monoclonal nonspecific suppressor factor *β* on tumor necrosis factor-*α* secretion in lipopolysaccharide-stimulated macrophages. [88] In this study, quercetin and the heat shock protein HSC70 together modulated the role of monoclonal nonspecific inhibitors *β*. In the macrophage-like cell line RAW264.7, quercetin dose-dependent inhibition of LPS/interferon *γ*-induced nitric oxide production without cytotoxicity. In addition, quercetin inhibits TNF-*α* enhancement and the production of RANTES, a member of the C-C chemokine superfamily in RAW264.7 cells. Quercetin may negatively control the function of MNSF*β* by modulating the action of the chaperone HSC70. Quercetin is effective in the treatment of knee osteoarthritis, and studies have shown that quercetin can alleviate joint damage in rats with knee osteoarthritis by mediating the TSC2-RHBE-mTOR signal pathway and that quercetin inhibits the expression of RHEB, p-mTOR, p-ULK1, and P62 and promotes fibroblast proliferation and migration. [91] Studies have shown that quercetin significantly reduces the expression level of TNF-*α*, IL-1*β,* and IL-6 in skin wounds, reduces inflammatory infiltration at the wound site, enhances the proliferation and migration of fibroblasts, inhibits inflammation in mice through Wnt/*β*-catenin signal pathway and TERT, reduces the level of inflammatory factors, and effectively promotes skin wound healing. [92].

Quercetin is known to suppress the activity of NF-*κ*B translocation, I-*κ*B-phosphorylation, AP-1, and reporter gene transcription and hence fights against inflammation. It also modulates the activity of NF-*κ*B, JNK1, and AP-1 signal pathways. The activity of TNF-*α* was also reduced when treated with quercetin [89]. Quercetin is a potent ferroptosis inhibitor and ameliorates acute kidney injury [93]. Quercetin exerts beneficial effects on type 2 diabetes potentially by inhibiting pancreatic iron deposition and pancreatic *β* cells ferroptosis [94]. Quercetin-3-O-glucuronide is a metabolite of quercetin that has been shown to block the role of renal platinum in tubular cells and can prevent tubular toxicity and play a renal protective role [95].

## 8. Conclusion

**Figure 3:**
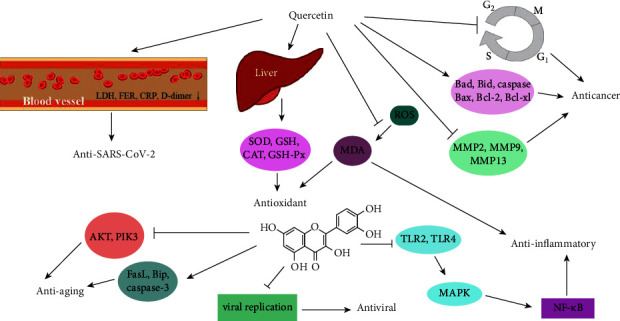
Pharmacological activity and the possible mechanisms of quercetin.

Many phytochemicals isolated from different natural sources have been thought to show therapeutic potential for many diseases; quercetin is one of the most popular and biologically active drugs among them. Through continuous research, quercetin is expected to become a new drug that can prevent and treat various diseases. A large number of *in vivo* and *in vitro* studies have shown that quercetin exhibits good pharmacological activities, including anti-SARS-CoV-2, antioxidant, anticancer, antiaging, antiviral, anti-inflammatory activities, and its possible related mechanisms as shown in Figure 3.

Although formulations of quercetin have been developed and partially used in clinical practice, there are still many questions to be solved due to its low bioavailability and low absorption. As far as the current research is concerned, the research on the pharmacological effect of quercetin is mainly concentrated in the nonclinical stage, and there is less clinical research on it, the absorption and metabolic mechanism of quercetin in the human body are not clear, before the pharmacological application, researchers need to further explore the pharmacological mechanism of quercetin in the human body in order to better apply to the prevention and treatment of clinical diseases.

## Figures and Tables

**Table 1:** Anticancer effects of quercetin and its mechanism.

Cancer types	Study designs	Quercetin/derivatives/sources	Study models	Effects/molecular mechanisms	Ref.
Prostate cancer	*In vitro*	Quercetin	PC3 and LNCaP cells	Increase cyt *c*, casp 3, casp 8, Bax, Bcl-2, p21Cip1, p27Kip1, and p53	[39]
				Decrease p38, NF-*κ*B, and survivin protein levels and increased the PTEN expression	
Liver cancer	*In vitro*	Quercetin	KIM-1, YN-1, KYN-2, YN-3, HAK-1A, HAK-1B, HAK-2, HAK-3, HAK-4, HAK-5, HAK-6 KMCH-1, and KMCH-2 cells	Inhibit apoptosis and cell cycle arrest	[43]
	*In vitro*	Quercetin	HepG2, HuH-6, and HepT1 cells. Human hepatoblastoma tissues	Promote the deacetylation of SIRT6 on FZD4 inhibit the activation of Wnt/*β*-catenin pathway	[47]
Hepatocellular carcinoma	*In vitro*	Quercetin	LM3 cells. Nude mice	Downregulation of the activation of JAK2 and STAT3	[48]
	*In vivo*				
Prostate cancer	*In vitro*	Quercetin	LNCaP, DU-145, and PC-3 cells	Affect the mitochondrial integrity and disturb the ROS homeostasis depending, activate Raf/MEK, AKT, and NF-*κ*B pathway	[61]
Colon carcinoma, androgen-sensitive cancer, prostate cancer, human myeloma, blood cancer, breast cancer, and ovarian cancer	*In vitro*	Quercetin	CT-26, PC-12, LNCaP, PC-3, MOLT-4, U266B1, raji, MCF-7, and CHO cells. Female BALB/c mice	Inhibit the growth of a panel of 9 cancer cell lines	[62]
	*In vivo*				
Human breast cancer	*In vitro*	Quercetin encapsulated in solid lipid nanoparticles	MCF-7 and MCF-10A cells	Increase (ROS) level and MDA contents, decrease antioxidant enzyme activity and expression of the Bcl-2 protein	[63]
Human breast cancer	*In vitro*	Quercetin, quercetin-3′-sulfate, and 1- quercetin-3-glucuronide	MCF-7 cells	Inhibit the growth of human breast cancer MCF-7 cells 19 in a dose-dependent manner, with IC_50_ values of 23.1, 27.6, and 73.2 *μ*M	[64]
Melanoma	*In vitro*	Quercetin	B164A5 cells	Reduce OCR and ECAR.	[65]
Pancreatic cancer	*In vitro*	Quercetin and BET inhibitors	K1, 8505c, MDA-T85, and CD18 cells	Decrease hnRNPA1 and enhance the effects of BET inhibitors at suppressing tumor growth	[66]

**Table 2:** Study of quercetin's *in vitro* and *in vitro*anti-inflammatory activity.

Disease types	Quercetin/derivatives	Study models	Effects/molecular mechanisms	Ref.
Endotoxin-induced inflammatory response	Quercetin and quercitrin	RAW264.7 cells	Reduce TNF-*α*, IL-1*β*, and IL-6	[4]
—	Quercetin	H9C2 cells	Promote PVT1 expression	[24]
Rheumatoid arthritis	Quercetin	Eight-week-old male C57BL/6 mice	Inhibit neutrophil infiltration	[82]
Surgical-induced osteoarthritis	Quercetin	New Zealand White rabbits	Upregulate SOD and TIMP-1, downregulate MMP-13, inhibiting the degradation of cartilage extracellular matrix	[85]
LPS-induced oxidative stress and inflammation	Quercetin	A549 cells	Suppress the nuclear translocation of NF-*κ*B, reduce levels of inflammatory cytokine tumor necrosis factor (TNF)-*α*, interleukin (IL)-1, and IL-6	[87]
LPS/interferon *γ*-induced nitric oxide production	Quercetin and heat shock protein HSC70	RAW264.7 cells	Inhibit enhancement of TNF-*α* and the production of RANTES	[88]
TNF-*α* induced inflammation	Quercetin	Human umbilical vein endothelial cells	Block NF-*κ*B and AP-1 signaling pathway	[89]
CCl_4_-induced inflammation	Quercetin	Male mice (20–25 g)	Inhibit ROS production in the liver and attenuate CCl_4_-induced oxidative damage	[90]

## Data Availability

All data used to support the findings of this study are included within the paper.

## References

[1] Ulusoy H. G., Sanlier N. (2020). A minireview of quercetin: from its metabolism to possible mechanisms of its biological activities. *Critical Reviews in Food Science and Nutrition*.

[2] Magar R. T., Sohng J. K. (2020). A review on structure, modifications and structure-activity relation of quercetin and its derivatives. *Journal of Microbiology and Biotechnology*.

[3] Nguyen T. L. A., Bhattacharya D. (2022). Antimicrobial activity of quercetin: an approach to its mechanistic principle. *Molecules*.

[4] Tang J., Diao P., Shu X., Li L., Xiong L. (2019). Quercetin and quercitrin attenuates the inflammatory response and oxidative stress in LPS-induced RAW264.7 cells: in vitro assessment and a theoretical model. *BioMed Research International*.

[5] Deepika M. P. K., Maurya P. K. (2022). Health benefits of quercetin in age-related diseases. *Molecules*.

[6] Deng Q., Li X. X., Fang Y., Chen X., Xue J. (2020). Therapeutic potential of quercetin as an antiatherosclerotic agent in atherosclerotic cardiovascular disease: a review. *Evidence-based Complementary and Alternative Medicine*.

[7] Kim D. H., Khan H., Ullah H. (2019). MicroRNA targeting by quercetin in cancer treatment and chemoprotection. *Pharmacological Research*.

[8] Heřmánková E., Zatloukalová M., Biler M. (2019). Redox properties of individual quercetin moieties. *Free Radical Biology and Medicine*.

[9] Zou H., Ye H., Kamaraj R., Zhang T., Zhang J., Pavek P. (2021). A review on pharmacological activities and synergistic effect of quercetin with small molecule agents. *Phytomedicine*.

[10] Akyuz E., Paudel Y. N., Polat A. K., Dundar H. E., Angelopoulou E. (2021). Enlightening the neuroprotective effect of quercetin in epilepsy: from mechanism to therapeutic opportunities. *Epilepsy and Behavior*.

[11] Karimi A., Naeini F., Asghari Azar V. (2021). A comprehensive systematic review of the therapeutic effects and mechanisms of action of quercetin in sepsis. *Phytomedicine*.

[12] Islam M. S., Quispe C., Hossain R. (2021). Neuropharmacological effects of quercetin: a literature-based review. *Frontiers in Pharmacology*.

[13] Dahiya R., Mohammad T., Roy S. (2019). Investigation of inhibitory potential of quercetin to the pyruvate dehydrogenase kinase 3: towards implications in anticancer therapy. *International Journal of Biological Macromolecules*.

[14] Chen B. L., Wang L. T., Huang K. H., Wang C. C., Chiang C. K., Liu S. H. (2014). Quercetin attenuates renal ischemia/reperfusion injury via an activation of AMP-activated protein kinase-regulated autophagy pathway. *The Journal of Nutritional Biochemistry*.

[15] Zhang T., Zhong S., Li T., Zhang J. (2018). Saponins as modulators of nuclear receptors. *Critical Reviews in Food Science and Nutrition*.

[16] Zhang J., Pavek P., Kamaraj R., Ren L., Zhang T. (2021). Dietary phytochemicals as modulators of human pregnane X receptor. *Critical Reviews in Food Science and Nutrition*.

[17] Agrawal P. K., Agrawal C., Blunden G. (2020). Quercetin: antiviral significance and possible COVID-19 integrative considerations. *Natural Product Communications*.

[18] Chen K., Shen Z., Wang G. (2022). Research progress of CRISPR-based biosensors and bioassays for molecular diagnosis. *Frontiers in Bioengineering and Biotechnology*.

[19] Derosa G., Maffioli P., D’Angelo A., Di Pierro F. (2021). A role for quercetin in coronavirus disease 2019 (COVID-19). *Phytotherapy Research*.

[20] Boretti A. (2021). Quercetin supplementation and COVID-19. *Natural Product Communications*.

[21] Di Pierro F., Iqtadar S., Khan A. (2021). Potential clinical benefits of quercetin in the early stage of COVID-19: results of a second, pilot, randomized, controlled and open-label clinical trial. *International Journal of General Medicine*.

[22] Colunga Biancatelli R. M. L., Berrill M., Catravas J. D., Marik P. E. (2020). Quercetin and vitamin C: an experimental, synergistic therapy for the prevention and treatment of SARS-CoV-2 related disease (COVID-19). *Frontiers in Immunology*.

[23] Aucoin M., Cooley K., Saunders P. R. (2020). The effect of quercetin on the prevention or treatment of COVID-19 and other respiratory tract infections in humans: a rapid review. *Advances in Integrative Medicine*.

[24] Li F., Liu J., Tang S. (2021). Quercetin regulates inflammation, oxidative stress, apoptosis, and mitochondrial structure and function in H9C2 cells by promoting PVT1 expression. *Acta Histochemica*.

[25] Xu D., Hu M. J., Wang Y. Q., Cui Y. L. (2019). Antioxidant activities of quercetin and its complexes for medicinal application. *Molecules*.

[26] Liu S., Zhu Y., Liu N., Fan D., Wang M., Zhao Y. (2021). Antioxidative properties and chemical changes of quercetin in fish oil: quercetin reacts with free fatty acids to form its ester derivatives. *Journal of Agricultural and Food Chemistry*.

[27] Tsai C. F., Chen G. W., Chen Y. C. (2021). Regulatory effects of quercetin on M1/M2 macrophage polarization and oxidative/antioxidative balance. *Nutrients*.

[28] Oh W. Y., Ambigaipalan P., Shahidi F. (2021). Quercetin and its ester derivatives inhibit oxidation of food, LDL and DNA. *Food Chemistry*.

[29] Arslan A. S., Seven I., Mutlu S. I., Arkali G., Birben N., Seven P. T. (2022). Potential ameliorative effect of dietary quercetin against lead-induced oxidative stress, biochemical changes, and apoptosis in laying Japanese quails. *Ecotoxicology and Environmental Safety*.

[30] Zhu X., Li N., Wang Y. (2017). Protective effects of quercetin on UVB irradiation-induced cytotoxicity through ROS clearance in keratinocyte cells. *Oncology Reports*.

[31] Saw C. L. L., Guo Y., Yang A. Y. (2014). The berry constituents quercetin, kaempferol, and pterostilbene synergistically attenuate reactive oxygen species: involvement of the Nrf2-ARE signaling pathway. *Food and Chemical Toxicology*.

[32] Babenkova I. V., Osipov A. N., Teselkin Y. O. (2018). The effect of dihydroquercetin on catalytic activity of iron (II) ions in the fenton reaction. *Bulletin of Experimental Biology and Medicine*.

[33] Dong B., Shi Z., Dong Y. (2022). Quercetin ameliorates oxidative stressinduced cell apoptosis of seminal vesicles via activating Nrf2 in type 1 diabetic rats. *Biomedicine & Pharmacotherapy*.

[34] Rauf A., Imran M., Khan I. A. (2018). Anticancer potential of quercetin: a comprehensive review. *Phytotherapy Research*.

[35] Reyes-Farias M., Carrasco-Pozo C. (2019). The anti-cancer effect of quercetin: molecular implications in cancer metabolism. *International Journal of Molecular Sciences*.

[36] Tang S. M., Deng X. T., Zhou J., Li Q. P., Ge X. X., Miao L. (2020). Pharmacological basis and new insights of quercetin action in respect to its anti-cancer effects. *Biomedicine & Pharmacotherapy*.

[37] Almatroodi S. A., Alsahli M. A., Almatroudi A. (2021). Potential therapeutic targets of quercetin, a plant flavonol, and its role in the therapy of various types of cancer through the modulation of various cell signaling pathways. *Molecules*.

[38] Neamtu A. A., Maghiar T. A., Alaya A. (2022). A comprehensive view on the quercetin impact on colorectal cancer. *Molecules*.

[39] Erdogan S., Turkekul K., Dibirdik I. (2018). Midkine downregulation increases the efficacy of quercetin on prostate cancer stem cell survival and migration through PI3K/AKT and MAPK/ERK pathway. *Biomedicine & Pharmacotherapy*.

[40] Maleki Dana P., Sadoughi F., Asemi Z., Yousefi B. (2021). Anti-cancer properties of quercetin in osteosarcoma. *Cancer Cell International*.

[41] Tavana E., Mollazadeh H., Mohtashami E. (2020). Quercetin: a promising phytochemical for the treatment of glioblastoma multiforme. *BioFactors*.

[42] Shrestha R., Mohankumar K., Martin G. (2021). Flavonoids kaempferol and quercetin are nuclear receptor 4A1 (NR4A1, Nur77) ligands and inhibit rhabdomyosarcoma cell and tumor growth. *Journal of Experimental & Clinical Cancer Research*.

[43] Hisaka T., Sakai H., Sato T. (2020). Quercetin suppresses proliferation of liver cancer cell lines in vitro. *Anticancer Research*.

[44] Vafadar A., Shabaninejad Z., Movahedpour A. (2020). Quercetin and cancer: new insights into its therapeutic effects on ovarian cancer cells. *Cell & Bioscience*.

[45] Brito A. F., Ribeiro M., Abrantes A. M. (2016). New approach for treatment of primary liver tumors: the role of quercetin. *Nutrition and Cancer*.

[46] Di Petrillo A., Orru G., Fais A., Fantini M. C. (2022). Quercetin and its derivates as antiviral potentials: a comprehensive review. *Phytotherapy Research*.

[47] Liu T., Li Z., Tian F. (2021). Quercetin inhibited the proliferation and invasion of hepatoblastoma cells through facilitating SIRT6-medicated FZD4 silence. *Human & Experimental Toxicology*.

[48] Wu L., Li J., Liu T. (2019). Quercetin shows anti-tumor effect in hepatocellular carcinoma LM3 cells by abrogating JAK2/STAT3 signaling pathway. *Cancer Medicine*.

[49] Teekaraman D., Elayapillai S. P., Viswanathan M. P., Jagadeesan A. (2019). Quercetin inhibits human metastatic ovarian cancer cell growth and modulates components of the intrinsic apoptotic pathway in PA-1 cell line. *Chemico-Biological Interactions*.

[50] Wu T.-C., Chan S.-T., Chang C.-N., Yu P.-S., Chuang C.-H., Yeh S.-L. (2018). Quercetin and chrysin inhibit nickel-induced invasion and migration by downregulation of TLR4/NF-*κ*B signaling in A549 cells. *Chemico-Biological Interactions*.

[51] Zhao J., Fang Z., Zha Z. (2019). Quercetin inhibits cell viability, migration and invasion by regulating miR-16/HOXA10 axis in oral cancer. *European Journal of Pharmacology*.

[52] Li S., Pei Y., Wang W., Liu F., Zheng K., Zhang X. (2019). Quercetin suppresses the proliferation and metastasis of metastatic osteosarcoma cells by inhibiting parathyroid hormone receptor 1. *Biomedicine & Pharmacotherapy*.

[53] Rather R. A., Bhagat M. (2020). Quercetin as an innovative therapeutic tool for cancer chemoprevention: molecular mechanisms and implications in human health. *Cancer Medicine*.

[54] Soofiyani S. R., Hosseini K., Forouhandeh H. (2021). Quercetin as a novel therapeutic approach for lymphoma. *Oxidative Medicine and Cellular Longevity*.

[55] Mansourizadeh F., Alberti D., Bitonto V. (2020). Efficient synergistic combination effect of Quercetin with Curcumin on breast cancer cell apoptosis through their loading into Apo ferritin cavity. *Colloids and Surfaces B: Biointerfaces*.

[56] Jia L., Huang S., Yin X., Zan Y., Guo Y., Han L. (2018). Quercetin suppresses the mobility of breast cancer by suppressing glycolysis through Akt-mTOR pathway mediated autophagy induction. *Life Sciences*.

[57] Xu W., Xie S., Chen X., Pan S., Qian H., Zhu X. (2021). Effects of quercetin on the efficacy of various chemotherapeutic drugs in cervical cancer cells. *Drug Design, Development and Therapy*.

[58] Sunoqrot S., Al-Debsi T., Al-Shalabi E. (2019). Bioinspired polymerization of quercetin to produce a curcumin-loaded nanomedicine with potent cytotoxicity and cancer-targeting potential in vivo. *ACS Biomaterials Science & Engineering*.

[59] Zang X., Cheng M., Zhang X., Chen X. (2021). Quercetin nanoformulations: a promising strategy for tumor therapy. *Food & Function*.

[60] Ezzati M., Yousefi B., Velaei K., Safa A. (2020). A review on anti-cancer properties of Quercetin in breast cancer. *Life Sciences*.

[61] Ward A. B., Mir H., Kapur N., Gales D. N., Carriere P. P., Singh S. (2018). Quercetin inhibits prostate cancer by attenuating cell survival and inhibiting anti-apoptotic pathways. *World Journal of Surgical Oncology*.

[62] Hashemzaei M., Far A. D., Yari A. (2017). Anticancer and apoptosisinducing effects of quercetin in vitro and in vivo. *Oncology Reports*.

[63] Niazvand F., Orazizadeh M., Khorsandi L., Abbaspour M., Mansouri E., Khodadadi A. (2019). Effects of quercetin-loaded nanoparticles on MCF-7 human breast cancer cells. *Medicina (Kaunas)*.

[64] Wu Q., Needs P. W., Lu Y., Kroon P. A., Ren D., Yang X. (2018). Different antitumor effects of quercetin, quercetin-3’-sulfate and quercetin-3-glucuronide in human breast cancer MCF-7 cells. *Food & Function*.

[65] Sturza A., Pavel I., Ancușa S. (2018). Quercetin exerts an inhibitory effect on cellular bioenergetics of the B164A5 murine melanoma cell line. *Molecular and Cellular Biochemistry*.

[66] Pham T. N. D., Stempel S., Shields M. A. (2019). Quercetin enhances the anti-tumor effects of BET inhibitors by suppressing hnRNPA1. *International Journal of Molecular Sciences*.

[67] Passos J. F., Saretzki G., Von Zglinicki T. (2007). DNA damage in telomeres and mitochondria during cellular senescence: is there a connection?. *Nucleic Acids Research*.

[68] Ogrodnik M., Miwa S., Tchkonia T. (2017). Cellular senescence drives age-dependent hepatic steatosis. *Nature Communications*.

[69] Pathak S., Regmi S., Nguyen T. T. (2018). Polymeric microsphere-facilitatedsite-specific delivery of quercetin prevents senescence of pancreatic islets in vivo and improves transplantation outcomes in mouse model of diabetes. *Acta Biomaterialia*.

[70] Wu L., Zhang Q., Mo W. (2017). Quercetin prevents hepatic fibrosis by inhibiting hepatic stellate cell activation and reducing autophagy via the TGF-*β*1/Smads and PI3K/Akt pathways. *Scientific Reports*.

[71] Hohmann M. S., Habiel D. M., Coelho A. L., Verri W. A., Hogaboam C. M. (2019). Quercetin enhances ligand-induced apoptosis in senescent idiopathic pulmonary fibrosis fibroblasts and reduces lung fibrosis in vivo. *American Journal of Respiratory Cell and Molecular Biology*.

[72] Kim S. R., Jiang K., Ogrodnik M. (2019). Increased renal cellular senescence in murine high-fat diet: effect of the senolytic drug quercetin. *Translational Research*.

[73] Wang T., Feng X., Li L. (2022). Effects of quercetin on tenderness, apoptotic and autophagy signalling in chickens during post-mortem ageing. *Food Chemistry*.

[74] Shakerian E., Afarin R., Akbari R., Mohammadtaghvaei N. (2022). Effect of Quercetin on the fructose-activated human hepatic stellate cells, LX-2, an in-vitro study. *Molecular Biology Reports*.

[75] Ohmae S., Akazawa S., Takahashi T., Izumo T., Rogi T., Nakai M. (2022). Quercetin attenuates adipogenesis and fibrosis in human skeletal muscle. *Biochemical and Biophysical Research Communications*.

[76] Hwang H. V., Tran D. T., Rebuffatti M. N., Li C. S., Knowlton A. A. (2018). Investigation of quercetin and hyperoside as senolytics in adult human endothelial cells. *PLoS One*.

[77] Wu W., Li R., Li X. (2015). Quercetin as an antiviral agent inhibits influenza A virus (IAV) entry. *Viruses*.

[78] Liu M., Yu Q., Xiao H. (2020). The inhibitory activities and antiviral mechanism of medicinal plant ingredient quercetin against grouper iridovirus infection. *Frontiers in Microbiology*.

[79] Kim C. H., Kim J. E., Song Y. J. (2020). Antiviral activities of quercetin and isoquercitrin against human herpesviruses. *Molecules*.

[80] Wang J., Hao K., Yu F. (2022). Field application of nanoliposomes delivered quercetin by inhibiting specific hsp70 gene expression against plant virus disease. *Journal of Nanobiotechnology*.

[81] Yang D., Wang T., Long M., Li P. (2020). Quercetin: its main pharmacological activity and potential application in clinical medicine. *Oxidative Medicine and Cellular Longevity*.

[82] Yuan K., Zhu Q., Lu Q. (2020). Quercetin alleviates rheumatoid arthritis by inhibiting neutrophil inflammatory activities. *The Journal of Nutritional Biochemistry*.

[83] Luo X., Bao X., Weng X. (2022). The protective effect of quercetin on macrophage pyroptosis via TLR2/Myd88/NF-*κ*B and ROS/AMPK pathway. *Life Sciences*.

[84] Saccon T. D., Nagpal R., Yadav H. (2021). Senolytic combination of dasatinib and quercetin alleviates intestinal senescence and inflammation and modulates the gut microbiome in aged mice. *The Journals of Gerontology: Series A*.

[85] Wei B., Zhang Y., Tang L., Ji Y., Yan C., Zhang X. (2019). Protective effects of quercetin against inflammation and oxidative stress in a rabbit model of knee osteoarthritis. *Drug Development Research*.

[86] Saeedi-Boroujeni A., Mahmoudian-Sani M. R. (2021). Anti-inflammatory potential of Quercetin in COVID-19 treatment. *Journal of Inflammation*.

[87] Sul O. J., Ra S. W. (2021). Quercetin prevents LPS-induced oxidative stress and inflammation by modulating NOX2/ROS/NF-kB in lung epithelial cells. *Molecules*.

[88] Nakamura M., Fukuma Y., Notsu K., Kono M. (2022). Quercetin and HSC70 coregulate the anti-inflammatory action of the ubiquitin-like protein MNSF*β*. *Molecular Biology Reports*.

[89] Chen T., Zhang X., Zhu G. (2020). Quercetin inhibits TNF-*α* induced HUVECs apoptosis and inflammation via downregulating NF-kB and AP-1 signaling pathway in vitro. *Medicine*.

[90] Ma J. Q., Li Z., Xie W. R., Liu C. M., Liu S.-S. (2015). Quercetin protects mouse liver against CCl4-induced inflammation by the TLR2/4 and MAPK/NF-*κ*B pathway. *International Immunopharmacology*.

[91] Lv S., Wang X., Jin S., Shen S., Wang R., Tong P. (2022). Quercetin mediates TSC2-RHEB-mTOR pathway to regulate chondrocytes autophagy in knee osteoarthritis. *Gene*.

[92] Mi Y., Zhong L., Lu S. (2022). Quercetin promotes cutaneous wound healing in mice through Wnt/*β*-catenin signaling pathway. *Journal of Ethnopharmacology*.

[93] Wang Y., Quan F., Cao Q. (2021). Quercetin alleviates acute kidney injury by inhibiting ferroptosis. *Journal of Advanced Research*.

[94] Li D., Jiang C., Mei G. (2020). Quercetin alleviates ferroptosis of pancreatic beta cells in type 2 diabetes. *Nutrients*.

[95] Munoz-Reyes D., Casanova A. G., Gonzalez-Paramas A. M. (2022). Protective effect of quercetin 3-O-glucuronide against cisplatin cytotoxicity in renal tubular cells. *Molecules*.

